# Getting “Just Deserts” or Seeing the “Silver Lining”: The Relation between Judgments of Immanent and Ultimate Justice

**DOI:** 10.1371/journal.pone.0101803

**Published:** 2014-07-18

**Authors:** Annelie J. Harvey, Mitchell J. Callan

**Affiliations:** Department of Psychology, University of Essex, Essex, United Kingdom; Brock University, Canada

## Abstract

People can perceive misfortunes as caused by previous bad deeds (immanent justice reasoning) or resulting in ultimate compensation (ultimate justice reasoning). Across two studies, we investigated the relation between these types of justice reasoning and identified the processes (perceptions of deservingness) that underlie them for both others (Study 1) and the self (Study 2). Study 1 demonstrated that observers engaged in more ultimate (vs. immanent) justice reasoning for a “good” victim and greater immanent (vs. ultimate) justice reasoning for a “bad” victim. In Study 2, participants' construals of their bad breaks varied as a function of their self-worth, with greater ultimate (immanent) justice reasoning for participants with higher (lower) self-esteem. Across both studies, perceived deservingness of bad breaks or perceived deservingness of ultimate compensation mediated immanent and ultimate justice reasoning respectively.

## Introduction

A long history of research into the psychology of justice and deservingness has demonstrated that people are motivated to make sense of and find meaning in their own and others' experiences of suffering and misfortune [1], [2], [3], and they do so in a variety of ways [4], [5], [6]. For example, on the one hand, people may attempt to perceive a “silver lining” in someone's undeserved suffering by adopting the belief that although a victim is currently suffering, she will ultimately be compensated for her misfortune [3]. In other words, through *ultimate justice reasoning*, people are able to extend the temporal framework of an injustice, such that any negative outcome previously endured will be ultimately compensated with a positive outcome. Research has confirmed that perceiving benefits in the later lives of victims of misfortunes is one way observers cognitively manage the threat imposed when observing undeserved suffering [7], [8], [9], [10]. For example, Anderson and colleagues found that participants, whose belief in a just world had been previously threatened, displayed a tendency to see a teenager's later life as more enjoyable and meaningful if he had been badly injured than if he suffered only a mild injury [7].

On the other hand, people may try to make sense of suffering and misfortune by engaging in *immanent justice reasoning* [11], [12], [13], for a review see [14], which involves causally attributing a negative outcome to someone's prior misdeeds, even if such a causal connection is illogical. For example, Callan and colleagues found that participants causally related a freak car accident to a man's prior behavior to a greater extent when they learned he stole from children than when he did not steal [15]. Immanent justice reasoning, then, allows an observer to maintain a perception of deservingness by locating the *cause* of a random misfortune in the prior misdeeds of the victim [11], [15], [14]. Indeed, research has shown that people engage in greater immanent justice reasoning when their justice concerns are heightened by first focusing on their long-term goals [15], cf. [16] or after being exposed to an unrelated instance of injustice [11].

Although research has shown that people readily engage in immanent and ultimate justice reasoning in response to suffering and misfortune, much less is known about how these responses interact and how they operate. Indeed, only a handful of studies have thus far examined ultimate and immanent justice reasoning simultaneously [17], [18], [19], and have primarily done so in the context of assessing individual differences in these justice beliefs. Understanding how these different reactions to misfortune operate not only informs future theorizing see [1], but also carries practical implications in predicting how people will react to victims in different circumstances. Thus, we sought to extend the literature on immanent and ultimate justice reasoning in three important ways: (1) by investigating whether there is a relation between immanent and ultimate justice reasoning, (2) by identifying the underlying processes that give rise to this relation, and (3) by examining whether immanent and ultimate justice reasoning operate the same way when people consider their own misfortune as when they consider the misfortunes of others (Study 2).

### The relation between immanent and ultimate justice reasoning

Maes and colleagues [18], [19] identified that people's individual endorsement of immanent and ultimate justice reasoning resulted in opposite reactions to victims. That is, people who believe strongly in ultimate justice reasoning are more likely to positively evaluate victims of misfortune, whereas people scoring highly in immanent justice beliefs blamed and derogated a victim for their plight. As immanent and ultimate justice reasoning are associated with conflicting victim reactions, these reactions to injustice may have a negative relation, such that the adoption of one form of justice reasoning reduces the extent to which people engage in the other. In Study 1, we sought to test this negative relation between these two types of justice reasoning empirically by assessing how people make sense out of misfortunes. We predicted that when people are given to ultimate justice reasoning (i.e., when the victim is a good person; see [7]), they would be less likely to engage in immanent justice reasoning. When people are given to immanent justice reasoning (i.e., when the victim is a bad person; see [14]), however, they would be less likely to perceive ultimate justice. We propose that the relation between the worth of the victim and justice reasoning is at least partly due to people's perceptions of what is considered as deserved.

### Perceived deservingness and immanent and ultimate justice reasoning

Responding to instances of suffering and misfortune with ultimate and immanent justice reasoning can be considered seemingly irrational. Although there may be logical reasons why good and bad people will have good or bad lives (e.g., higher well-being from a good person acting prosocially), often no substantial causal links exist between a person's character, their random misfortune, and their ultimate fulfillment in life; or a victim's previous misdeeds and their current misfortune. That is, the worth of a person does not cause random, unrelated misfortunes and enduring a random misfortune does not necessarily mean that an individual's later life will be better. Despite this seeming irrationality, people might nonetheless engage in immanent and ultimate justice reasoning in response to suffering and misfortune because doing so enables them to maintain important, functional beliefs. We examined whether immanent and ultimate justice reasoning might be driven, in part, by the belief that the world is a just, fair, and nonrandom place where people get what they deserve—a world where an appropriate relation exists between the value of people (good or bad) and the value of their outcomes (good or bad) [20], [3], see also [21]. In other words, both the processes of causally linking a random misfortune to someone's prior misdeeds (immanent justice) and perceiving benefits in the later lives of victims of misfortune (ultimate justice) might be driven, in part, by a concern for upholding notions of *deservingness*.

Deservingness refers to the perceived congruence between the value of a person and the value of his or her outcomes. Therefore, something bad happening to a “good” person is often perceived as undeserved, whereas the same outcome occurring to a “bad” person is often considered deserved [11], [22], [21], [23], [24]. Several studies have confirmed that the perceived deservingness of a random outcome is an important mediator of the extent to which people are willing to adopt immanent justice accounts of the outcome see [14]. Less is known, however, about the processes underlying ultimate justice reasoning. If the proposed negative relation between immanent and ultimate justice reasoning is driven by the ultimate goal of perceiving people's fates as deserved in a just world, we predict that perceived deservingness should underlie the endorsement of both types of justice reasoning.

This analysis is consistent with Kruglanski's discussion of the principle of equifinality [25], which suggests that different substitutable and equal means are capable of reaching the same goal. In the context of the current research, immanent and ultimate justice reasoning can both be considered equal means to achieving the goal of preserving a belief that the world is a fair and just place where people get what they deserve. People can accomplish this goal via immanent justice reasoning by attributing the cause of a misfortune to the victim's prior misdeeds. Alternatively, people who engage in ultimate justice reasoning can uphold their just-world beliefs by believing that a victim's misfortune will be ultimately compensated [7]. If participants engage in one type of reasoning because of their concerns about deservingness, utilizing an additional type of reasoning would be redundant. For example, linking an individual's current misfortune to their prior misdeeds satisfies a concern for deservingness because the victim “got what she deserved”. Further rationalizations of misfortune, such as believing the victim will be ultimately compensated, are therefore less necessary and support our prediction of a negative correlation between ultimate and immanent justice reasoning.

The extent to which perceived deservingness underlies immanent and ultimate justice reasoning, however, should depend on the specific outcome people believe is deserved. With immanent justice reasoning, causal connections are drawn between people's previous deeds and their *recently experienced outcomes*, whereas ultimate justice reasoning entails believing in more *“long-term” positive outcomes* for a victim who is suffering. Thus, whether a concern for deservingness helps explain immanent and ultimate justice reasoning should depend on what people perceive as deserved—later life fulfillment or a recently experienced random outcome—given the value of the person experiencing the outcome. The idea that specific perceptions of deservingness might differentially predict immanent and ultimate justice reasoning resonates well with research showing greater congruency between constructs that are measured at the same level of specificity (e.g., values and behavior) [26]. Accordingly, we examined the degree to which perceptions of deserving later-life fulfillment and a recently experienced outcome underlie ultimate and immanent justice reasoning, respectively. We predicted that perceiving a misfortune as deserved should better predict immanent justice reasoning [14], whereas perceiving a victim as deserving of later fulfillment should better predict ultimate justice reasoning.

### Immanent and ultimate justice reasoning for the self

Lerner argued that principles of justice and deservingness for others should be equivalent to the self, as observing deservingness in another's life should mean, by generalization, that one's own life is just and fair [3], [27]. Early work by Lerner and colleagues [28], [29] showed that people are more likely to work towards fairness for others when they themselves have received unfair treatment, suggesting that people are responsive to the fates of others because this determines the fairness of the world they live in. As a result, one's own fate “is intertwined emotionally and practically with the ability of others to get what they deserve” [28] (p. 177).

Consistent with this view, observer judgments of deservingness are often comparable to deservingness judgments made for the self. That is, research has shown that people judge others, and themselves, as deserving bad (good) outcomes if they are perceived as bad (good) people [11], [22], [30], [23], [24], [31], [32]. For example, Wood and colleagues found that individuals chronically and situationally lower (vs. higher) in self-esteem saw themselves as more deserving of negative emotions [31]. More recently, Callan and colleagues found that participants' beliefs about deserving bad outcomes in life mediated the relation between trait self-esteem and a variety of self-defeating thoughts and behaviors (e.g., self-handicapping, thoughts of self-harm) [22]. Although this research highlights the important role that perceptions of deservingness for the self play in a host of self-relevant outcomes, no research to our knowledge has examined the role that personal deservingness plays in people's immanent justice and ultimate justice reasoning for self-relevant outcomes. To this end, in Study 2 we examined whether people would causally attribute their random bad breaks to their personal worth or believe they would achieve a fulfilling life as a function of their self-esteem and perceptions of deservingness. In other words, we examined whether the same relation between immanent and ultimate justice reasoning, and the same underlying processes of deservingness, in response to the misfortune of others (Study 1) would replicate when individuals considered their own misfortune (Study 2).

### Current research

Over two sets of studies we sought to investigate whether (1) there is a negative relation between immanent and ultimate justice reasoning, (2) perceived deservingness underlies this relation, and (3) the relation and processes involved in immanent and ultimate justice reasoning are similar for one's own misfortunes as they are for the misfortunes of others. To accomplish these aims we manipulated the worth of a victim (Study 1) or measured people's perceived self-worth (Study 2) before assessing judgments of deservingness and ultimate and immanent justice reasoning.

If there is a negative relation between immanent and ultimate justice reasoning in response to misfortune, then people should engage in significantly more ultimate than immanent justice reasoning for a victim who is a good person and significantly more immanent than ultimate justice reasoning for a victim who is a bad person. We also predicted that specific perceptions of deservingness would underlie this relation, such that perceiving a victim as deserving of their misfortune would more strongly mediate immanent justice reasoning and perceiving a victim as deserving of a fulfilling later life would more strongly mediate ultimate justice reasoning. Finally, we predicted that this pattern of findings should be similar when participants consider their own misfortunes (Study 2).

## Study 1

In Study 1 we manipulated the value of a victim of misfortune before assessing participants' perceptions of the degree to which he deserved his misfortune and deserved ultimate compensation along with immanent and ultimate justice reasoning. We predicted that a “good” victim would encourage participants to engage in more ultimate than immanent justice reasoning, largely due to the victim being deserving of ultimate compensation following their ill fate. When faced with a “bad” victim, however, we predicted that participants would interpret the victim's fate as deserved and therefore engage in more immanent rather than ultimate justice reasoning.

### Method

#### Participants

The study was administrated online and approved by the Ethics Committee of the University of Essex. Consent was achieved by asking participants to click a button to begin the study and give their consent or to close their browser and withdraw consent. We recruited two samples of participants (*Ns* = 168 and 100; total *N* = 268, 48.9% females, 0.4% unreported; *M*_age_ = 35.35, *SD*_age_ = 11.88) via Amazon's Mechanical Turk [33] and CrowdFlower. Twelve participants (4.5%) who incorrectly answered a simple manipulation question (“Is Keith Murdoch awaiting trial for sexually assaulting a minor?”) were excluded from further analysis. The samples differed only in the ordering of the items (see procedure below).

#### Materials and procedure

Participants were told they would be partaking in a study “investigating memory and impressions of events”. Participants were first presented with an ostensibly real news article that described a freak accident where a volunteer swim coach, Keith Murdoch, was seriously injured following a tree collapsing on his vehicle during high winds see [15]. Next, we manipulated the worth of the victim by telling participants that the victim was either a pedophile (“bad” person) or a respected swim coach (“good” person). Specifically, participants in the “bad” person condition learned that “Keith Murdoch is awaiting trial for sexually assaulting a 14-year-old boy while he worked at the Bitterne Leisure Center as a volunteer swim coach and that other charges of sexual exploitation of minors are pending given recent evidence obtained by police since the original charge.” Participants in the “good” person condition read that “Keith Murdoch volunteered as a swimming coach at the Bitterne Leisure Centre and is a valued and beloved member of the community.” We predicted that this information about the victim's character should determine how deserving the victim was of his random misfortune and ultimate compensation and, as a result, the extent of participants' immanent and ultimate justice reasoning respectively.

As a manipulation check, participants rated the goodness of the victim's character with the item, “How would you rate Keith Murdoch as a person?” (1 =  *very bad* to 6 =  *very good*).

*Ordering of items for Sample 1.* In our first sample, participants were then asked two questions to assess their perceptions of deservingness of the accident: “To what extent do you feel Keith Murdoch deserved to be in this accident?” (1 =  not at all deserving to 6 =  very deserving) and “To what extent do you feel that this accident was a just and fair outcome for Keith Murdoch?” (1 =  not at all just and fair to 6 =  very just and fair).

Adapted from items used to measure beliefs in conspiracy theories [34], participants then answered four items that assessed their immanent justice attributions for the accident: “To what extent do you feel it is worth considering that this accident might have been a result of Keith Murdoch's conduct as a swim coach?” (1 =  *not at all worth considering* to 6 =  *worth considering*), “How possible do you feel it is that this accident was a result of Keith Murdoch's conduct as a swim coach?” (1 =  *not at all possible* to 6* =  possible*), “How plausible do you feel it is that this accident was caused by Keith Murdoch's conduct as a swim coach?” (1 =  *not at all plausible* to 6 =  *plausible*), and “I feel that this accident was a result of Keith Murdoch's conduct as a swim coach.” (1 =  *strongly disagree* to 6 =  *strongly agree*).

Following this, participants were asked two items to assess perceptions of how deserving the victim was of ultimate compensation: “I feel that Keith Murdoch deserves to experience his life as meaningful in the long run” and “I believe Keith Murdoch deserves to find purpose and fulfillment later in his life” (1 =  *strongly disagree* to 6 =  *strongly agree*). Finally, three items assessed ultimate justice reasoning see [7]: “To what extent do you think Keith Murdoch will find his existence fulfilling later in his life?”, “To what extent do you believe that in the future, Keith Murdoch will experience his life as meaningful?”, and “To what extent do you think that in the long run, Keith Murdoch will find purpose in his life?” (1 =  *not at all fulfilling/meaningful/purposeful* to 6 =  *very fulfilling/meaningful/purposeful*). All items within each construct reached acceptable internal consistency (see Table 1), so were averaged to form measures of perceived deservingness of an accident, immanent justice attributions, perceived deservingness of ultimate compensation, and ultimate justice judgments.

**Table 1:** Descriptive and inferential statistics for the measures employed in Studies 1 and 2.

	Worth of Victim Manipulation				Intercorrelations				
Measures	Pedophile	Volunteer	*t*	*d*	1.	2.	3.	4.	5.
**Study 1**	*M (SD)*	*M (SD)*							
1. Deservingness of misfortune	3.26 (1.65)	1.34 (0.71)	12.19**	1.51	[.86]	-	-	-	-
2. Immanent justice reasoning	1.98 (1.34)	1.27 (0.70)	5.28**	0.66	.56**	[.94]	-	-	-
3. Deservingness of later fulfillment	3.19 (1.29)	5.09 (0.73)	14.57**	1.81	-.67**	-.35**	[.86]	-	-
4. Ultimate justice reasoning	2.49 (1.08)	4.66 (0.97)	16.93**	2.11	-.64**	-.33**	.76**	[.97]	-
**Study 2**	*M*	*SD*							
1. Self-esteem	4.46	0.98	-	-	[.93]	-	-	-	-
2. Deservingness of misfortune	2.81	1.18	-	-	-.36**	[.69]	-	-	-
3. Immanent justice reasoning	2.93	1.22	-	-	-.39**	.66**	[.94]	-	-
4. Deservingness of later fulfillment	4.81	0.95	-	-	.38**	-.23*	-.16*	[.68]	-
5. Ultimate justice reasoning	4.70	1.05	-	-	.62**	-.25**	-.22*	.43**	[.94]

*Note.* Higher values indicate more of each construct. Where applicable, alpha reliabilities (or the bivariate correlations for the deservingness measures) are presented in brackets.

*p<.05

**p<.01

*Ordering of items for Sample 2.* Because we were concerned that the fixed ordering of our items in Sample 1 may have biased participants toward the first opportunity they were given to resolve the injustice (i.e., immanent justice reasoning), we recruited another sample of participants and reversed the ordering of items from Sample 1. Sample 2, therefore, was identical to Sample 1, with the exception of the ordering of items. The questionnaire was structured so that after rating the goodness of the victim's character, participants answered the items regarding how deserving the victim was of ultimate compensation and deserving of the accident, followed by the ultimate justice reasoning items and finally the immanent justice reasoning items.

### Results and Discussion

Preliminary analyses showed that there were no significant differences between the two samples in terms of the effect of the experimental manipulation on our dependent measures or the correlations among the measures (i.e., there were no significant interactions with sample/item order, all *p*s>.05), and the same patterns of results replicated across samples. Thus, the ordering of items did not appear to affect participants' responses. Accordingly, data from the two samples were collated and analyzed together.

Analysis of the manipulation check confirmed that participants who learned that the victim was a pedophile (*M* = 1.64, *SD* = 0.76) perceived him as less good than participants who learned that he was a respected volunteer (*M* = 5.14, *SD* = 0.57), *t*(251) = 41.66, *p*<.001, *d* = 5.22). Shown in Table 1, participants who were presented with a “bad” victim rated him as more deserving of his random bad outcome than participants who read about a “good” victim, conceptually replicating previous research [11], [35]. Also, participants who were presented with a “good” victim saw him as more deserving of later fulfillment than a “bad” victim. Table 1 also shows the correlations among the measures we employed in Study 1. Of note, both types of perceived deservingness correlated significantly with both types of justice judgments, and immanent and ultimate justice reasoning correlated negatively.

#### The interplay between immanent and ultimate justice reasoning

To examine the interplay between immanent and ultimate justice reasoning as a function of the value of the victim, we conducted a 2 (victim worth: good vs. bad) by 2 (type of justice reasoning: immanent justice vs. ultimate justice) mixed model ANOVA, with type of justice reasoning as the within-subjects factor. Because people are typically more willing to endorse ultimate justice than immanent justice in absolute terms, we standardized the data for comparisons across types of justice reasoning (the unstandardized data is presented in Table 1). Analyses revealed the predicted Victim Worth X Type of Reasoning interaction, *F*(1, 254) = 176.09, *p*<.001, η*_p_* ^2^ = .41.

**Figure 1:**
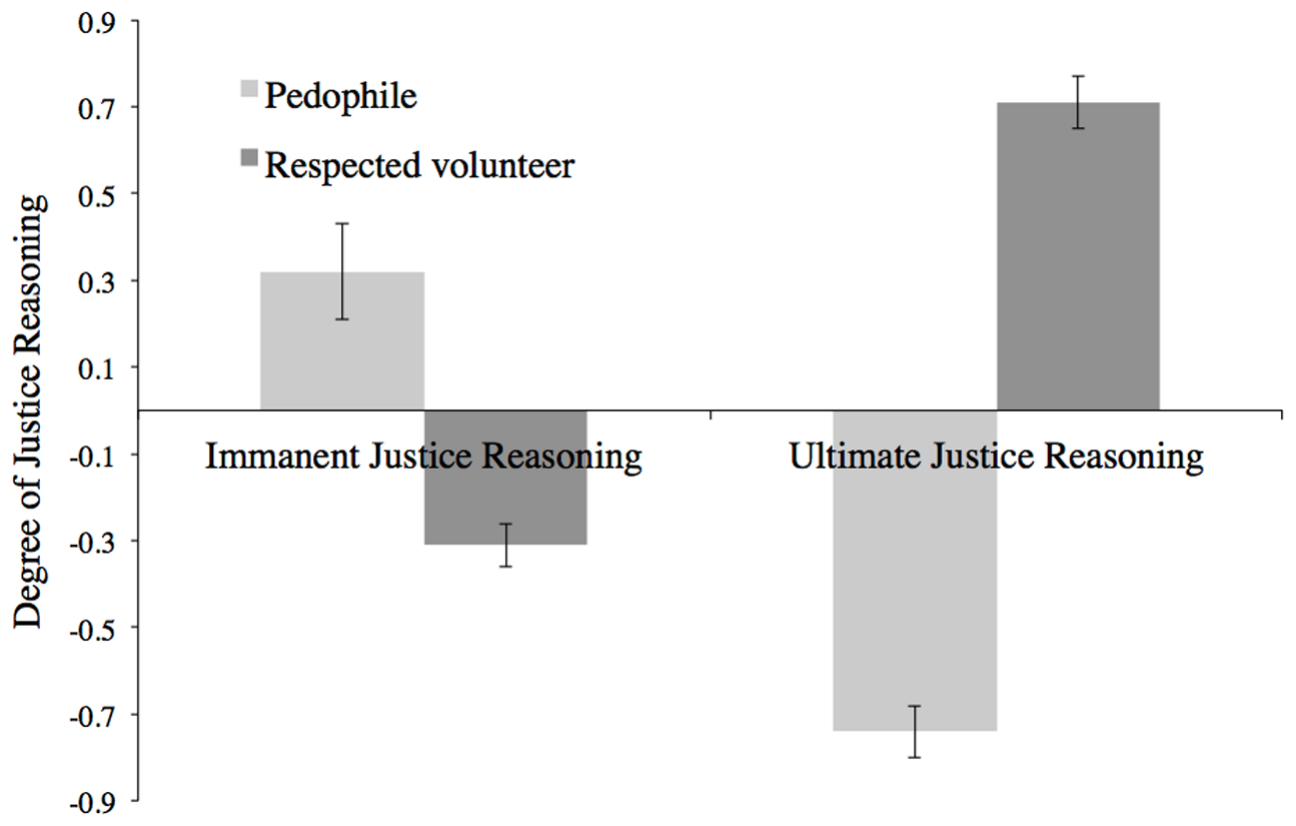
Mean level of immanent justice and ultimate justice reasoning from Study 1 (standardized) as a function of the victim's personal worth (pedophile versus respected volunteer). Error bars show standard errors of the means.

Shown in Figure 1, decomposing the interaction revealed that participants engaged in relatively more immanent justice than ultimate justice reasoning when the victim was a pedophile, *t*(124) = 7.96, *p*<.001, and more ultimate justice than immanent justice reasoning when he was a respected volunteer, *t*(130) = 12.01, *p*<.001.

#### Perceived Deservingness

We examined whether the perceived deservingness of the victim's fate accounts for the observed relation between participants' judgments of immanent justice and ultimate justice. That is, a concern for deservingness should underpin the degree to which people engage in more or less immanent justice reasoning relative to ultimate justice reasoning as a function of the worth of the victim. More specifically, perceiving a victim as deserving of his fate should better underlie immanent justice judgments and perceiving a victim as deserving of later life fulfillment should better predict ultimate justice reasoning, as a function of the victim's worth.

**Figure 2:**
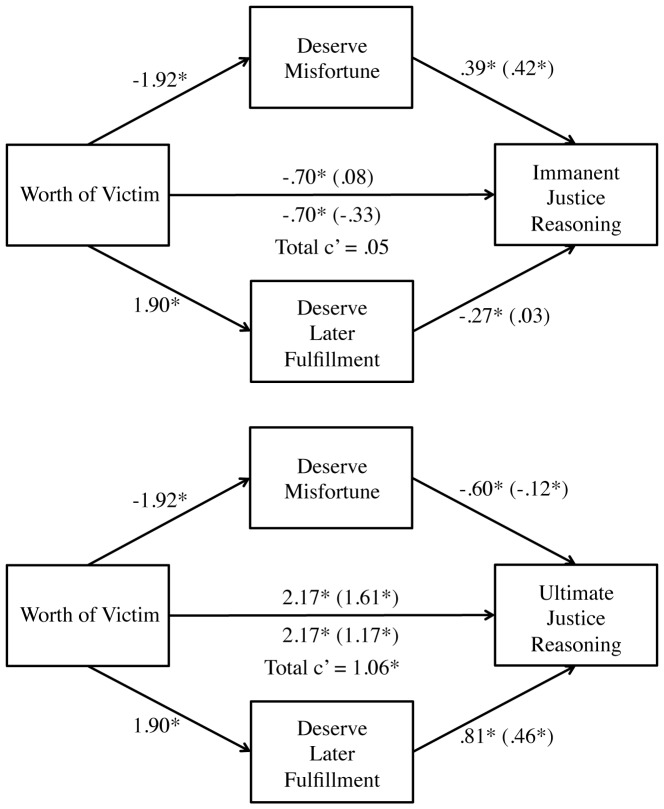
Mediational model from Study 1, predicting immanent justice and ultimate justice reasoning from the worth of a victim, beliefs about deserving bad outcomes, and beliefs about deserving later fulfillment. The victim of negative worth (pedophile) was coded as 1 and the victim of positive worth (respected volunteer) was coded as 2. Values show unstandardized path coefficients. * *p*<.05.

To test this hypothesis, we conducted multiple mediation analyses with Preacher and Hayes's (2008) bootstrapping procedure (10,000 resamples; see Figure 2) [36]. As predicted, bootstrapping analyses revealed that perceived deservingness of the accident mediated the effect of the victim's worth on immanent justice reasoning (indirect effect  = −0.81, BCa CI = −1.13 to −0.56), but perceived deservingness of later fulfillment did not (indirect effect  = 0.06, BCa CI  = −0.19 to 0.31). The same analysis conducted with ultimate justice reasoning showed both types of deservingness mediated the effect of the victim's worth on justice reasoning, but perceived deservingness of later fulfillment (indirect effect  = .88, BCa CI = 0.63 to 1.15) was a stronger mediator than perceived deservingness of the accident (indirect effect  = .23, BCa CI  = .06 to 0.45). The same mediation pattern was observed for both samples separately. The exception being that for the second sample, perceived deservingness of the accident did not mediate the effect of the manipulation on ultimate justice reasoning (cf. Study 2; indirect effect  = −0.02, BCa CI = −0.24 to 0.25). In sum, the value of a victim affects whether people view the misfortune or later life fulfillment as deserved, which in turn predicts the extent of immanent justice reasoning over ultimate justice reasoning and vice versa.

## Study 2

In Study 2, we sought to conceptually replicate our Study 1 findings in the context of participants' considerations of their *own* misfortunes. Study 1 found that participants perceived greater immanent justice for a victim with negative (vs. positive) worth and greater ultimate justice reasoning for a victim of positive (vs. negative) worth. In Study 2, we predicted that people's perceived self-worth should similarly influence the extent of justice reasoning for their own outcomes. Specifically, we assessed whether people are more likely to engage in immanent or ultimate justice reasoning for the self after considering their own misfortunes as a function of their perceptions of personal deservingness. To test this notion, we measured participants' self-esteem before asking them to respond to deservingness, immanent, and ultimate justice items in relation to their own recent bad breaks. Paralleling our Study 1 effects, we predicted that self-esteem would correlate negatively with immanent justice reasoning and positively with ultimate justice reasoning. Crucially, we predicted that perceived deservingness would underlie the relations between self-esteem and justice reasoning for the self. Per our Study 1 findings, we predicted that perceiving a bad break as deserved would better predict immanent justice reasoning for the self and perceiving oneself as deserving of later life fulfillment should be a better predictor of ultimate justice judgments for the self.

### Method

#### Participants

Participants were recruited online via Amazon's Mechanical Turk for a nominal payment (*N* = 102) or the University of Essex volunteer e-mail list for the chance to win a £20 gift voucher (*N* = 100; total *N* = 202, 56.9% females; *M*_age_ = 27.64, *SD*_age_ = 9.58). One participant was excluded from further analysis because he/she only answered one item from the self-esteem measure. Ethical approval and informed consent was obtained in the same way as Study 1.

#### Materials and procedure

Participants took part in a study that was ostensibly about “people's perceptions of their personal experiences.” We first assessed participant's self-esteem via Rosenberg's 10-item self-esteem scale (1 =  *strongly disagree* to 6 = *strongly agree*) [37]. We then asked participants to think about their recent random “bad breaks.” Bad breaks were described to participants as “those sorts of negative experiences we have that we do not intend, expect, or plan to occur—they just happen to us.”

Next, participants answered a questionnaire similar to that of Study 1, although the questions were framed around participants' personal random bad breaks and in more general terms, due to the recalled “bad breaks” being general events rather than a specific incident of victimization. First, participants answered two items that aimed to assess their perceived deservingness of general bad outcomes: “I often feel that I deserve the bad breaks that happen to me” and “When I've experienced bad breaks in my life, I've sometimes thought that I deserved them” (1 =  *strongly disagree* to 6 =  *strongly agree*). Similar items from Study 1 were used to assess immanent justice reasoning (e.g., “How possible do you feel it is that your bad breaks were a result of the kind of person you are?”). Next, we presented participants with two items that assessed how deserving they felt of greater life fulfillment and meaningfulness (e.g., “I feel that I deserve to experience my life as meaningful in the long run”) and three ultimate justice items based on those from Study 1 (e.g., “To what extent do you think you will find your existence fulfilling later in life?”). Table 1 shows that each of these measures achieved acceptable internal consistency.

### Results and Discussion

Shown in Table 1, participants' self-esteem was negatively related to immanent justice judgments, showing that the lower their self-esteem, the more participants felt their bad breaks were caused by the kind of person they were. Self-esteem and ultimate justice reasoning were positively related, indicating that the higher participants' self-esteem, the more they engaged in ultimate justice reasoning for themselves. These findings replicate our Study 1 results, but do so in the context of participants considering their *own* bad breaks rather than the misfortune of someone else. Indeed, reflecting the interaction pattern shown in Figure 1, a test of the difference between overlapping correlations [38] showed that the correlation between self-esteem and immanent justice reasoning was significantly different from the correlation between self-esteem and ultimate justice reasoning (95% confidence interval: −1.16, −.85).

Of particular importance was the mediating role of deservingness beliefs in these relations, which we specified into two forms: (1) the deservingness of past bad breaks and (2) the deservingness of later life fulfillment. We again conducted multiple mediation analyses with Preacher and Hayes's (2008) bootstrapping procedure (10,000 resamples) [36]. When entering both deservingness of bad breaks and deservingness of later fulfillment as possible mediators of the relation between self-esteem and immanent justice reasoning, only the former provided a significant indirect effect. In other words, perceived deservingness of bad breaks significantly mediated the relation between self-esteem and immanent justice reasoning (indirect effect  = −0.27, BCa CI = −0.41 to −0.14) but perceived deservingness of later fulfillment did not (indirect effect  = 0.03, BCa CI = −0.04 to 0.08). Conducting the same analysis for ultimate justice reasoning revealed that perceived deservingness of bad breaks did not mediate the relation between self-esteem and ultimate justice reasoning (indirect effect  = 0.003, BCa CI  = −0.05 to 0.06) but perceived deservingness of later life fulfillment did (indirect effect  = 0.09, BCa CI = 0.03 to 0.19).

**Figure 3:**
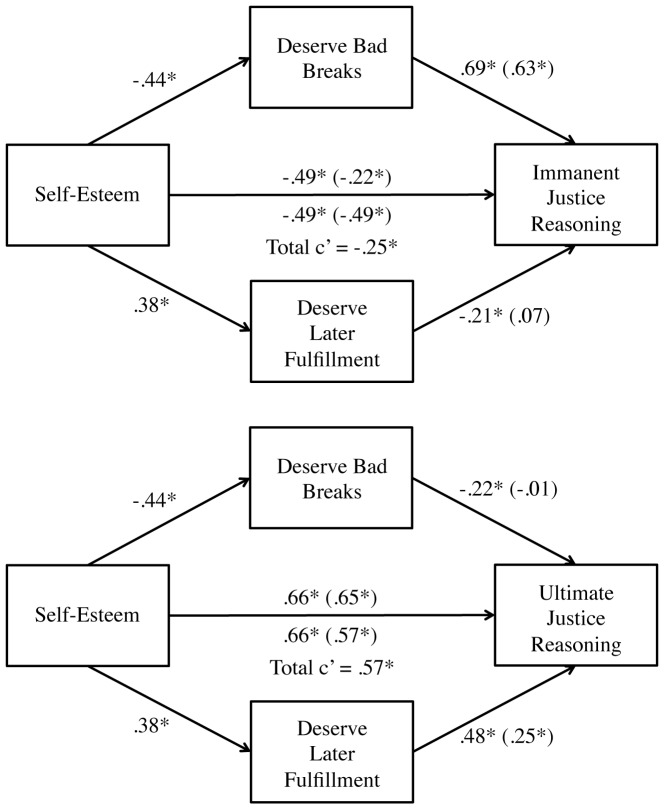
Mediational model from Study 2, predicting immanent justice and ultimate justice reasoning from self-esteem, beliefs about deserving bad outcomes, and beliefs about deserving later fulfillment. Values show unstandardized path coefficients. * *p*<.05.

Therefore, only deservingness of bad breaks mediated the relation between self-esteem and immanent justice reasoning, whereas only deservingness of later life fulfillment mediated the relation between self-esteem and ultimate justice reasoning for the self (see Figure 3).

## General Discussion

Over two studies we sought to determine (1) the relation between immanent justice and ultimate justice reasoning, (2) the underlying mechanism responsible for this relation, and (3) if the relation between immanent and ultimate justice reasoning not only applies to the misfortunes of others, but also to one's own misfortunes. Study 1 showed that participants engaged in immanent justice reasoning to a greater extent when they learned that a victim was a “bad” (vs. “good”) person, whereas they perceived more ultimate justice reasoning when the victim was a “good” (vs. “bad”) person. When people are given to making immanent justice attributions (i.e., when a victim is of low worth), ultimate justice judgments are lower. However, when individuals are prone to ultimate justice reasoning (i.e., when a victim is of high worth), immanent justice reasoning is reduced.

Importantly, perceived deservingness mediated these effects. When confronted with a “good” person who experienced a random ill-fate, participants saw the victim as deserving of later life fulfillment and therefore, rejected an immanent justice account of the event in favor of perceiving benefits in the later life of the victim. When the victim was considered in negative terms, however, participants were more willing to see the misfortune as deserved and causally attribute the freak accident to the victim's past behavior, as well as reducing their ultimate justice judgments accordingly. As a result, participants engaged in immanent and ultimate justice reasoning as a function of their concerns for deservingness. The type of perceived deservingness that best predicted the extent of justice reasoning was that which was the most compatible on specificity. In other words, perceived deservingness of the current misfortune was more specific to immanent justice reasoning and proved to be the strongest predictor. However, perceptions of deservingness in later life outcomes was more congruent with ultimate justice reasoning and therefore best predicted people's ultimate justice judgments.

Study 2 extended these findings into the domain of considering one's own bad breaks and future fulfillment in life. After thinking about their own bad breaks, ultimate justice reasoning for the self was greater among participants higher in self-esteem, whereas immanent justice reasoning was more pronounced among participants lower in self-esteem. Study 2 also mirrored Study 1′s effects of deservingness as underling these reactions to one's own outcomes. The perceived deservingness of bad breaks mediated the negative relation between self-esteem and immanent justice attributions, whereas only perceived deservingness of future life fulfillment mediated the positive relation between self-esteem and ultimate justice reasoning for the self.

These findings contribute to the literature in two important and novel ways: First, we examined how people try to make sense out of the misfortunes of others by engaging in both immanent and ultimate justice reasoning at once. We showed that these two types of justice reasoning are negatively related to one another and perceived deservingness plays an important role in the interplay between immanent and ultimate justice reasoning in response to the misfortunes of others. These findings therefore contribute to the limited literature examining when, and for whom, different reactions to instances of misfortune are apparent [1], [9], [17], [39], [40], [10]. As Hafer and Bègue argued, no one response is dominant across situations or individuals, and therefore multiple reactions should be assessed to gain a more comprehensive knowledge of how people make sense out of and find meaning in suffering and misfortune [1], also see [41]. Our work takes one step in that direction by suggesting the worth of a victim is key to determining perceptions of deservingness, which in turn influences the extent of both immanent and ultimate justice reasoning.

Of course, responding in terms of immanent and ultimate justice are by no means the only ways people make sense of misfortune and suffering. Interestingly, our manipulation of victim worth in Study 1 could be considered a manipulation of “just-world” threat, presumably because the “good” victim poses a larger threat to participants' just-world beliefs than the “bad” victim. Research has shown that people perceive the suffering of “good” victims as more unfair than the suffering of “bad” victims (e.g., when a physically attractive vs. an unattractive person is harmed) [42], [43], [44], [45]. Therefore, the interplay between other known responses to just-world threat, such as victim blaming see [1], and the responses to misfortune we measured here have yet to be investigated. It is therefore important for future research to examine perceptions of immanent and ultimate justice alongside other means by which people might maintain a perception of justice in the face of threat.

Second, the interactive pattern between the worth of a victim and type of justice reasoning we observed in Study 1 was replicated in Study 2 in the context of participants considering their own misfortunes. Of particular intrigue, we found that participants lower in self-esteem saw themselves as more deserving of their negative outcomes and were willing to adopt immanent justice attributions for their own fortuitous bad breaks. Although research into immanent justice reasoning has almost exclusively focused on people's causal attributions for the random misfortunes occurring to *others* [14], we found that the same processes operate when people entertain the causes of their own random bad breaks, and personal deservingness plays a crucial mediating role in this relation. In addition, we found that participants with higher self-esteem believed they were more deserving of, and would therefore receive, a fulfilling and meaningful life. These findings add to the existing literature on how people make sense of their misfortunes [46] by suggesting that perceived deservingness of ultimate compensation plays an important meditational role.

Further, our findings may be important and applicable to our understanding of people's coping and resilience in the face of personal suffering and misfortune. Some research has shown that sufferers of illnesses engage in thought processes akin to ultimate and immanent justice reasoning, and these types of reasoning can be either beneficial or detrimental to their health [47], [48], [49], [50]. Our findings suggest that deservingness—either in the form of deserving one's recent bad breaks or deserving fulfillment later in life—might be underlying these types of responses to misfortune and as a result, may determine the trajectory of patient's well-being and recovery. For example, believing that one contracted an illness because they were a bad person deserving of bad outcomes may lead to heightened anxiety, lower levels of life-satisfaction, and a reduced likelihood of recovery cf. [48]. In a similar vein, Callan and colleagues found that individuals who held stronger beliefs that they deserved bad outcomes engaged in more self-defeating behaviors, including self-handicapping, wanting close others to evaluate them negatively, and seeking negative feedback about their performance during an intelligence test [22]. On the other hand, adopting the belief that one deserves a fulfilling and meaningful life in the future may lead to greater general well-being in the face of illness cf. [47]. Of course, more research is needed on the role that these deservingness beliefs might play in people's responses to their own misfortunes, but our work offers a theoretical perspective and empirical findings that point to their potential importance.

Finally, the present research encourages related lines of future research. We considered immanent and ultimate justice as reactions to undeserved *negative* outcomes, but both of these types of justice reasoning might also be adopted when people make sense of undeserved *positive* outcomes e.g., [11]. Therefore, it is important for future research to extend these findings in the context of positive outcomes. Although some research has examined the effects of undeserved positive outcomes on immanent justice reasoning (e.g., a man won the lottery *because* he was pleasant and hard working) [11], to our knowledge no research has considered ultimate justice reasoning in response to undeserved positive outcomes. We speculate that observing a good person experiencing a good outcome should result in individuals perceiving the two as causally connected (i.e., immanent justice reasoning) cf. [11], but observing the same outcome occurring to a bad person should encourage individuals to believe that the lucky individual will receive their comeuppance in the future (i.e., ultimate justice reasoning). Although much of just-world research has been concerned with victims of misfortune see [1], Lerner suggested that any injustice, good or bad, threatens our commitment to a just world [27]. Therefore, to further our understanding of how responses to misfortune operate, it is important for future research to consider both sides of the coin—people's responses to undeserved positive outcomes as a well as undeserved negative outcomes.

## Supporting Information

Dataset S1 **Study 1.** Raw data and composite scores from Study 1 in SPSS.
(sav)

Dataset S2 **Study 2.** Raw data and composite scores from Study 2 in SPSS.
(sav)
